# Supplementary material for: Differential Expression of Amaranth AtrDODA Gene Family Members in Betalain Synthesis and Functional Analysis of AtrDODA1-1 Promoter
Source: Plants (Basel). 2025 Feb 4;14(3):454. doi: 10.3390/plants14030454 (PMC11821215; doi:10.3390/plants14030454)
Supplement: Supplementary file 1 [file plants-14-00454-s001.zip › S table S1 Nomenclature and basic physical and chemical properties of AtrDODA gene family member.pdf]

Supplemen table S1 Nomenclature and basic physical and chemical properties of *AtrDODA* gene family members in *Amaranthus*

Gene name	Gene ID	Number of amino acids	Molecular weight	Theoretical pl	Gravy	Instability index	Signal peptide	Subcellular localization
AtrDODA1-1	g23453.t1	286	32617.97	6.03	-0.460	43.48	No	Nucl
AtrDODA1-2	g14871.t1	268	30224.24	6.21	-0.385	34.45	No	Cyto and nucl
AtrDODA2-1	g14870.t1	269	30199.33	7.26	-0.368	46.85	No	Cyto and nucl

Note: Nucl: nucleus, Cyto: cytoplasm
