# Supplementary material for: Differential Expression of Amaranth AtrDODA Gene Family Members in Betalain Synthesis and Functional Analysis of AtrDODA1-1 Promoter
Source: Plants (Basel). 2025 Feb 4;14(3):454. doi: 10.3390/plants14030454 (PMC11821215; doi:10.3390/plants14030454)
Supplement: Supplementary file 1 [file plants-14-00454-s001.zip › S table S2 The Ka-Ks values of the collinear gene pairs of AtrDODA in Amaranthus tricolor.pdf]

Supplementary table S2 The Ka/Ks values of the collinear gene pairs of *AtrDODA* in amaranth

Gene name or Gene ID	Gene name or Gene ID	Ka	Ks	Ka/Ks
AtrDODA1-1	transcript:KMT13936	0.15	0.36	0.42
AtrDODA2-1	transcript:KMT18891	0.12	0.51	0.24
AtrDODA2-1	Bou_130730	0.16	0.56	0.29
AtrDODA2-1	Bou_74018	0.21	0.47	0.45
AtrDODA2-1	evm.model.LG06.1319	0.07	0.38	0.18
AtrDODA2-1	evm.model.LG18.1890	0.27	1.16	0.23
AtrDODA2-1	evm.model.LG20.27	0.13	0.54	0.24
AtrDODA2-1	AtrDODA1-2	0.14	0.42	0.33
