# Supplementary material for: Differential Expression of Amaranth AtrDODA Gene Family Members in Betalain Synthesis and Functional Analysis of AtrDODA1-1 Promoter
Source: Plants (Basel). 2025 Feb 4;14(3):454. doi: 10.3390/plants14030454 (PMC11821215; doi:10.3390/plants14030454)
Supplement: Supplementary file 1 [file plants-14-00454-s001.zip › S table S3 qRT-PCR primer sequence of related genes.pdf]

Supplementary table S3 qRT-PCR primer sequence of related genes

Primer name	Forward primer (5'→3')	Reverse primer (5'→3')	Primer use
AtrSAND	TCTTCAGAGTTCTCATCGCC	TCATCTCGTCGGTATTGGG	*Actin*
AtrDODA1-1	TGATTACCCTGATGCTATGTACC	AAACTGGGATGTCTGCTTGG	qPCR
AtrDODA2-1	TGGGCTGCTGAGTTTGATAA	GCTTGGAACGGTGAACTTGTA	qPCR
AtrDODA1-2	ATGATTTCTATGGCTTCCCTG	GGACACCTTCCTCCTTCAGA	qPCR
NtL25	CCCCTCACCACAGAGTCTGC	AAGGGTGTTGTTGTCCTCAATC TT	*Actin*
GUS	GTAGAAACCCCAACCCGTGAA	CGTAATGAGTGACCGCATCGA	qPCR
