# Supplementary material for: Differential Expression of Amaranth AtrDODA Gene Family Members in Betalain Synthesis and Functional Analysis of AtrDODA1-1 Promoter
Source: Plants (Basel). 2025 Feb 4;14(3):454. doi: 10.3390/plants14030454 (PMC11821215; doi:10.3390/plants14030454)
Supplement: Supplementary file 1 [file plants-14-00454-s001.zip › S table S4 Primer sequence of AtrDODA family related genes in Amaranth.pdf]

Supplementary table S4 Primer sequence of *AtrDODA* family related genes in *Amaranth*

Primer name	Forward primer (5'→3')	Reverse primer (5'→3')	Primer use
AtrDODA1-1	ACGCGTCGACATGAGTCCAACT GAAAATCC	CGGGATCCCGGTTGGAAGT GAACTTGTAGGCA	RT-PCR
AtrDODA2-1	ACGCGTCGACATGAGCGCAGGA AGAATTAA	CGGGATCCCGGCTTGGAAC GGTGAACTTGTA	RT-PCR
AtrDODA1-2	ACGCGTCGACATGGGTAGTCAA GAAATCATC	CGGAATTCGCTTGAAACAAA TTTGTAGGATCC	RT-PCR
Red-*AtrDODA 1-1*pro	ATCCTCTAGAGTCGACACCCAG TCGCACAAAAAAC	CTCAGATCTACCATGGTTTTCT CTTGAGTTTTTAAGGCAAAC	RT-PCR
Green-*AtrDO DA1-1*pro	ATCCTCTAGAGTCGACACCCAG TCGCACAATAAACC	CTCAGATCTACCATGGTTTTCT CTTGAGTTTTTAAGGCAAAC	RT-PCR
