# Supplementary figures and images for: Differential Expression of Amaranth AtrDODA Gene Family Members in Betalain Synthesis and Functional Analysis of AtrDODA1-1 Promoter
Source: Plants (Basel). 2025 Feb 4;14(3):454. doi: 10.3390/plants14030454 (PMC11821215; doi:10.3390/plants14030454)

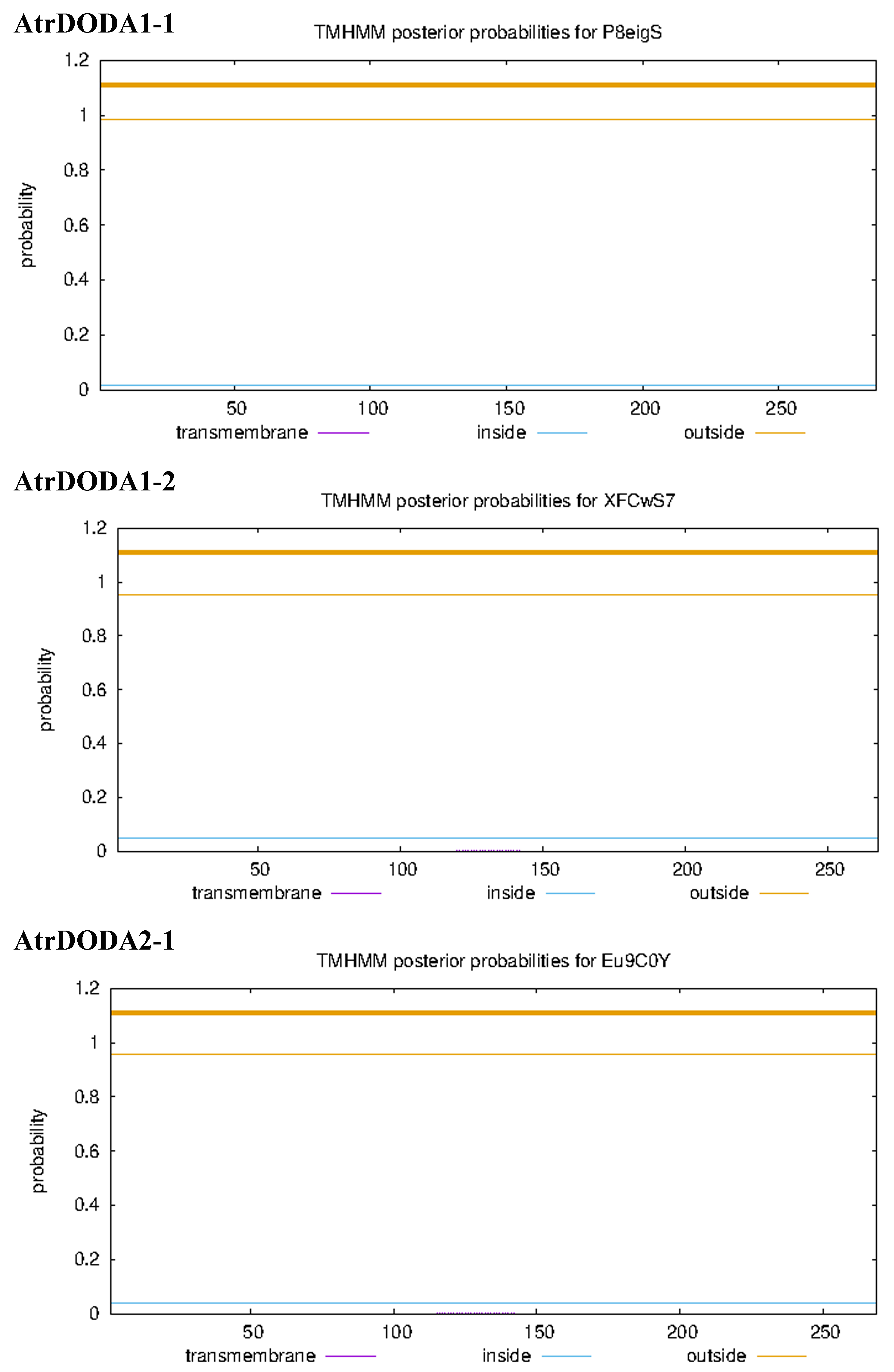

Supplement: Supplementary file 1 [file plants-14-00454-s001.zip › S Figure S1. Prediction of transmembrane structure of AtrDODAs proteins.jpg]

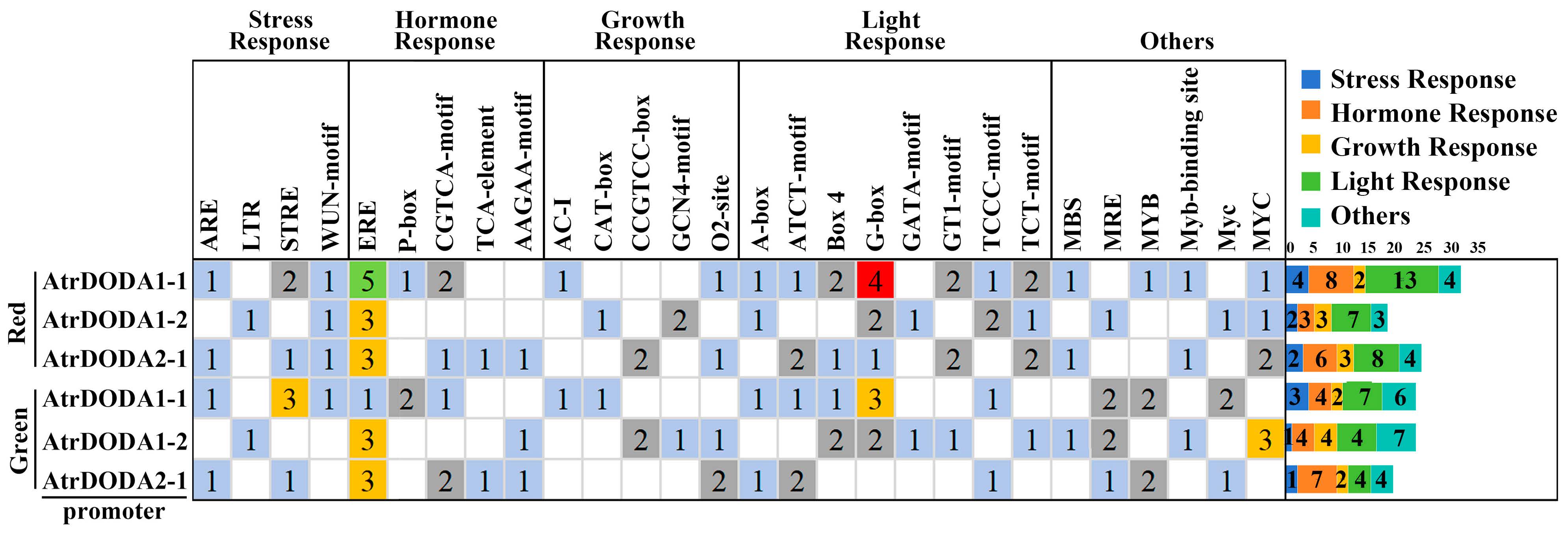

Supplement: Supplementary file 1 [file plants-14-00454-s001.zip › S Figure S2. Classification of different biological functions of cis-regulatory elem.jpg]

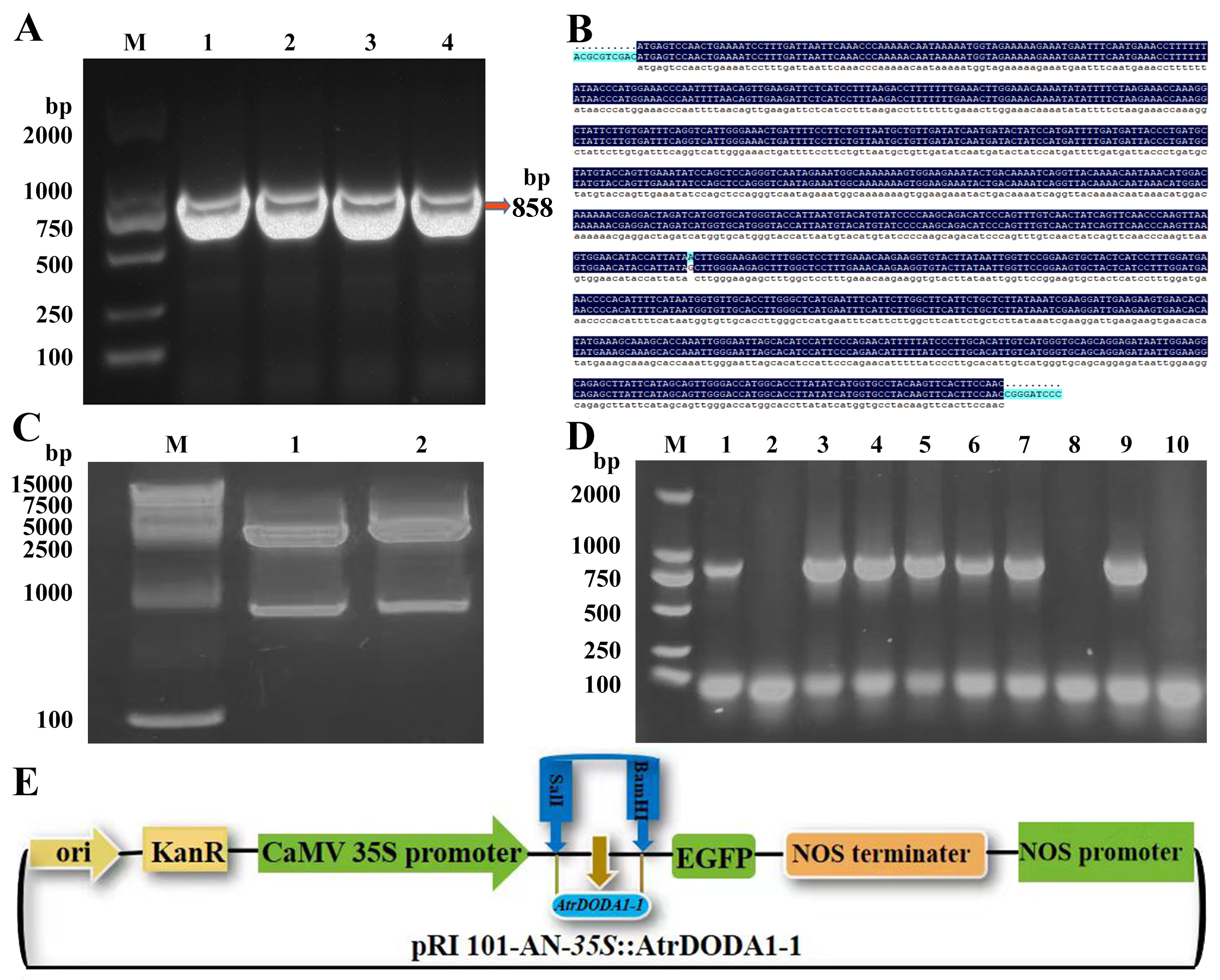

Supplement: Supplementary file 1 [file plants-14-00454-s001.zip › S Figure S3. Construction of the AtrDODA1-1 expression vector.jpg]

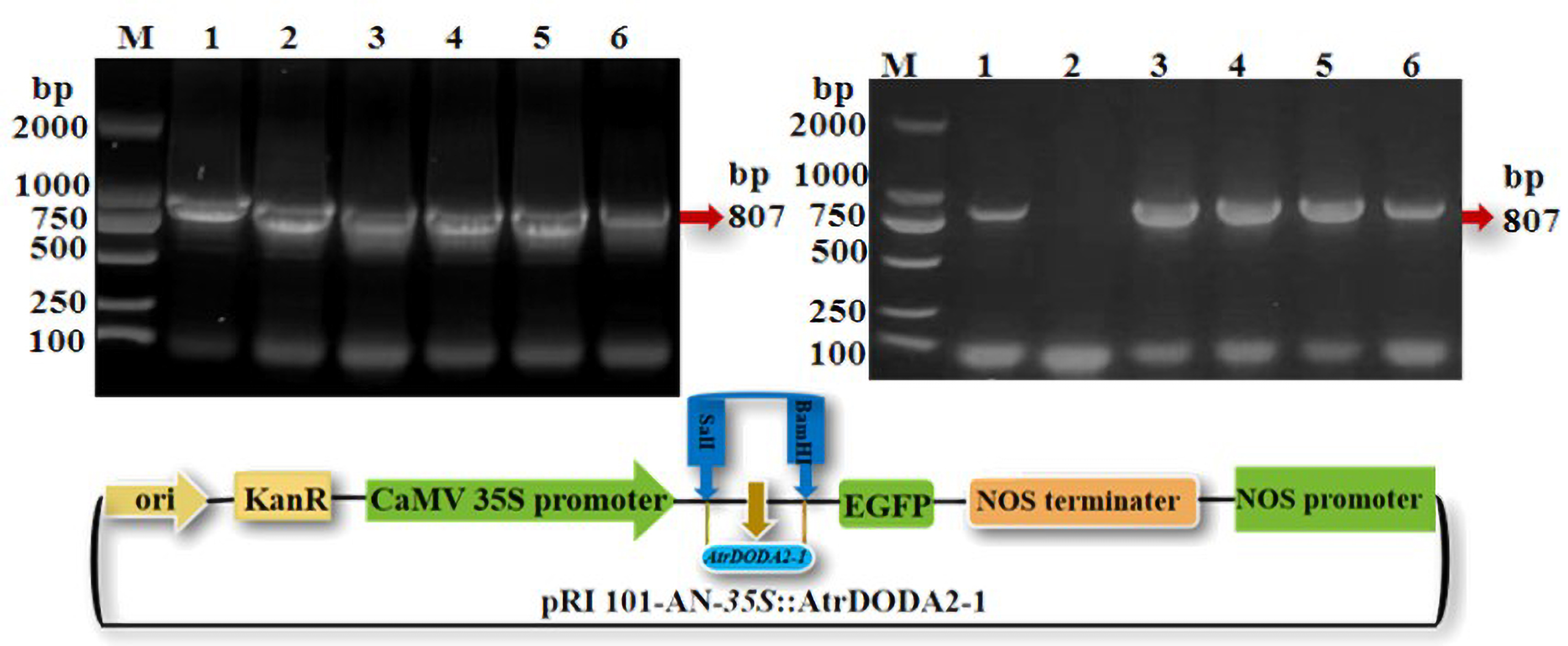

Supplement: Supplementary file 1 [file plants-14-00454-s001.zip › S Figure S4. Construction of the AtrDODA2-1 expression vector.jpg]

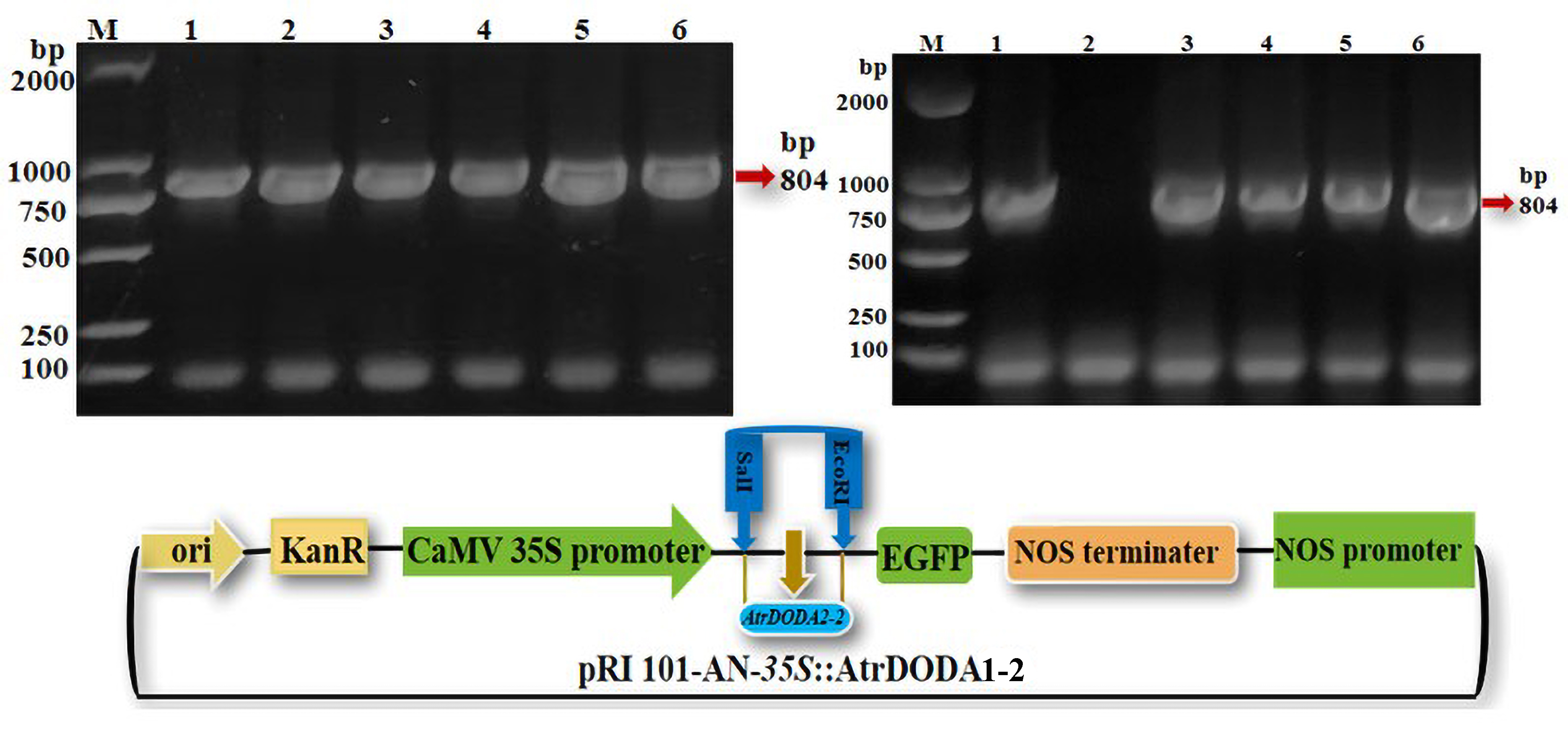

Supplement: Supplementary file 1 [file plants-14-00454-s001.zip › S Figure S5. Construction of the AtrDODA1-2 expression vector.jpg]

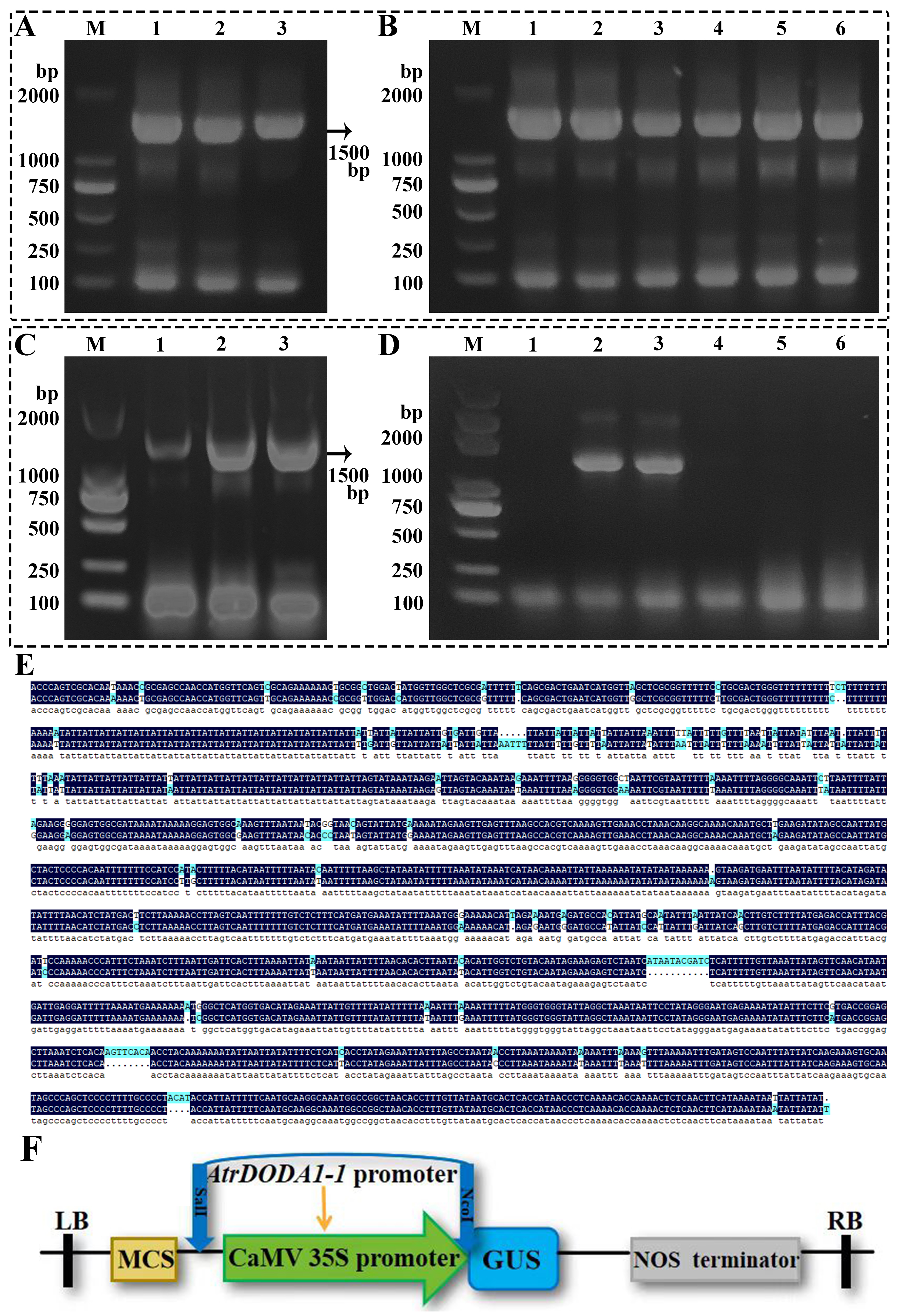

Supplement: Supplementary file 1 [file plants-14-00454-s001.zip › S Figure S6. Construction of promoter vectors for AtrDODA1-1 in red and green Amaranth.jpg]

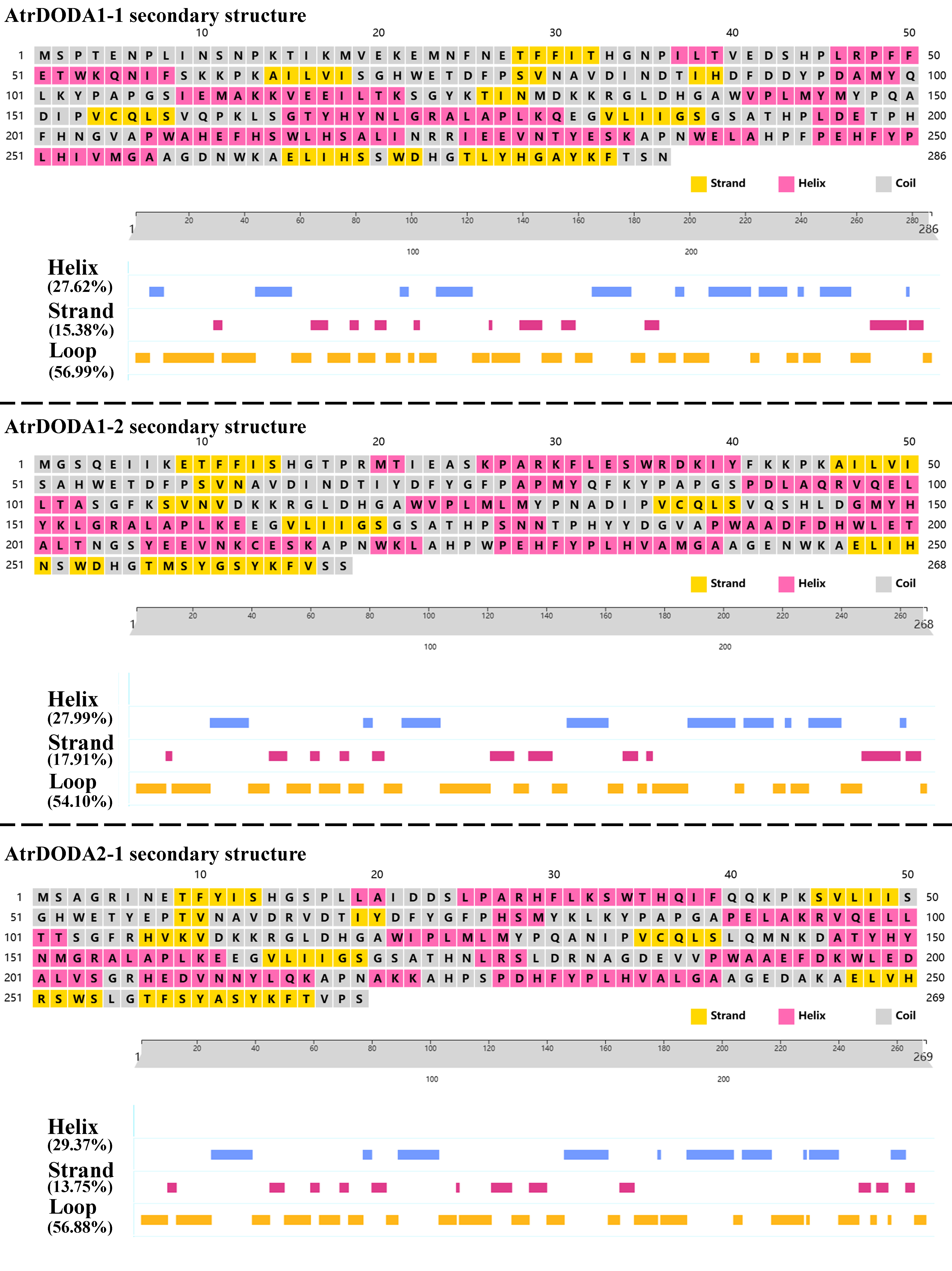

Supplement: Supplementary file 1 [file plants-14-00454-s001.zip › S Figure S7.Secondary structure of Amaranth AtrDODAs proteins.jpg]

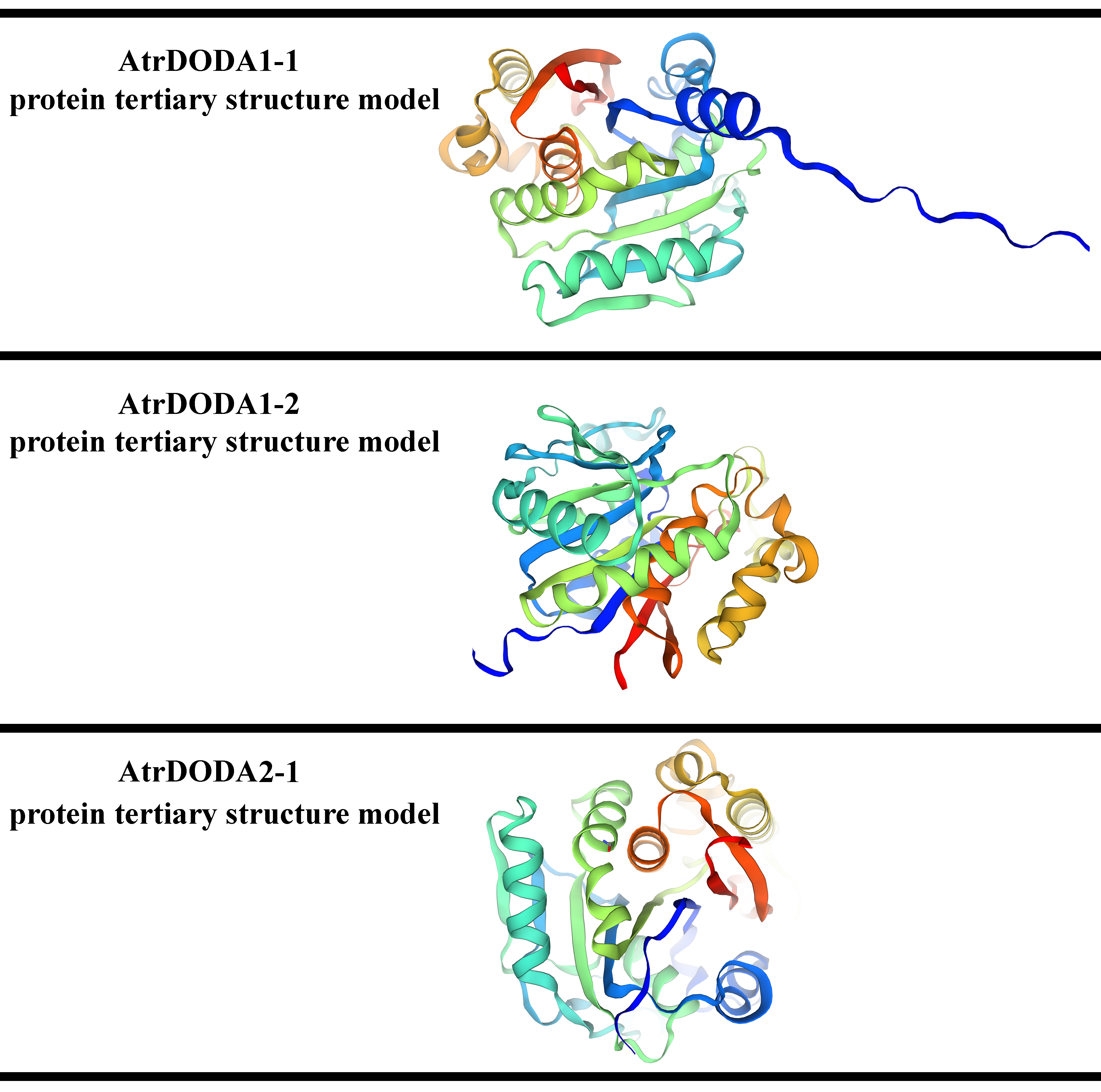

Supplement: Supplementary file 1 [file plants-14-00454-s001.zip › S Figure S8. Tertiary structure of Amaranth AtrDODAs proteins.jpg]
